# The Effectiveness of Pre-Operative Screening Tests in Determining Viral Infections in Patients Undergoing Oral and Maxillofacial Surgery

**DOI:** 10.3390/healthcare10071348

**Published:** 2022-07-20

**Authors:** Shintaro Sukegawa, Yuka Sukegawa, Kazuaki Hasegawa, Sawako Ono, Tomoya Nakamura, Ai Fujimura, Ayaka Fujisawa, Keisuke Nakano, Kiyofumi Takabatake, Hotaka Kawai, Yumika Mukainaka, Hitoshi Nagatsuka, Yoshihiko Furuki

**Affiliations:** 1 Department of Oral and Maxillofacial Surgery, Kagawa Prefectural Central Hospital, 1-2-1, Asahi-machi, Takamatsu 760-8557, Japan; yuka611225@gmail.com (Y.S.); de421040@s.okayama-u.ac.jp (K.H.); tmy.nakamura@s.okayama-u.ac.jp (T.N.); sugar.x.48@gmail.com (A.F.); ayafuji05@gmail.com (A.F.); furukiy@ma.pikara.ne.jp (Y.F.); 2 Department of Oral Pathology and Medicine, Graduate School of Medicine, Dentistry and Pharmaceutical Sciences, Okayama University, Okayama 700-8525, Japan; pir19btp@okayama-u.ac.jp (K.N.); gmd422094@s.okayama-u.ac.jp (K.T.); de18018@s.okayama-u.ac.jp (H.K.); mukainaka.y@gmail.com (Y.M.); jin@md.okayama-u.ac.jp (H.N.); 3 Department of Pathology, Kagawa Prefectural Central Hospital, Takamatsu 760-8557, Japan; de19008@s.okayama-u.ac.jp

**Keywords:** hepatitis B, hepatitis C, human immunodeficiency virus, pre-operative examination

## Abstract

We analyzed the rate of patients with hepatitis B virus (HBV), hepatitis C virus (HCV), or human immunodeficiency virus (HIV) infection diagnosed by pre-operative screening and estimated its cost. We retrospectively analyzed patients who underwent elective surgery at our maxillofacial surgery department between April 2014 and March 2022. We compared the number of patients with each infection identified by pre-operative screening and a pre-operative questionnaire. We also compared the prevalence of infections with varying age, sex, and oral diseases, and calculated the cost of screening per positive result. The prevalence of HBV, HCV, and HIV was 0.39% (62/15,842), 0.76% (153/15,839), and 0.07% (10/12,745), respectively. The self-reported rates were as follows: HBV, 63.4% (26/41); HCV, 50.4% (62/123); HIV, 87.5% (7/8). Differences in sex were statistically significant for all infectious diseases; age significantly affected HBV and HCV rates. There was no association between the odds ratio of oral disease and viral infections. The cost per positive result was $1873.8, $905.8, and $11,895.3 for HBV, HCV, and HIV, respectively. Although self-assessment using questionnaires is partially effective, it has inadequate screening accuracy. Formulating an auxiliary diagnosis of infectious diseases with oral diseases was challenging. The cost determined was useful for hepatitis, but not HIV.

## 1. Introduction

In 2011, 1.12–1.27 million people were estimated to have persistent hepatitis B virus (HBV) infection and 980,000 to 1.58 million people with persistent hepatitis C virus (HCV) infection in Japan [1]. The number of human immunodeficiency virus (HIV)-positive patients has also been increasing [2]. In recent years, testing for the hepatitis virus and HIV has become common in medical institutions as a pre-operative screening test for medical safety. Patients with needlestick injuries are always at risk of being infected with HBV, HCV, or HIV, especially during oral and maxillofacial surgery, where sharp appliances, such as arch bars and ligature wires or osteosynthesis plate and screws [3,4,5], are often used. In addition, oral and maxillofacial surgeons, who often contact the patient’s blood and saliva, may be exposed to pathogenic microorganisms, such as HIV and the hepatitis virus. Many researchers have reported a rate of 2–10 injuries/100 procedures [6,7,8]. The risk of infection with HIV, HBV, or HCV following a single needlestick injury varies widely, with HIV, HCV, and HBV at approximately 0.3, 3, and 30–50%, respectively [9,10]. Therefore, pre-operative screening for infectious diseases is important for oral and maxillofacial surgeons; moreover, a non-invasive screening is desirable.

However, patients may not be aware of being infected. Furthermore, although patient interviews and self-reported screenings are non-invasive and inexpensive, their effectiveness is unknown. Therefore, many Japanese institutions conduct blood tests to screen patients for infections before surgery. In Japan, as of 2022, the inspection fee for each infectious disease is 880 yen (approximately 7.3 US dollars (120 yen to the US dollar; 20 March 2022)) for HBV, 1050 yen (approximately 8.8 US dollars) for HCV, and 1120 yen (approximately 9.3 US dollars) for HIV. Although these tests are effective in determining a rapid post-exposure response, no scientific evidence exists for this expensive tool [9,10,11].

This study aimed to evaluate the prevalence and incidence of HBV, HCV, and HIV infection identified by pre-operative screening, and the subsequent testing costs. In addition, we investigated the self-reported positivity rate of patients for these infections and the prevalence of hepatitis and HIV infection in patients based on their age, sex, and the presence of oral diseases and conditions.

## 2. Materials and Methods

### 2.1. Study Design

This single-center retrospective study was conducted from April 2014 to March 2022 in the Department of Oral and Maxillofacial Surgery, Kagawa Prefectural Central Hospital, Japan. Institutional review board approval was obtained (approved number; 1110). The study was performed in accordance with the Declaration of Helsinki.

### 2.2. Patients

We retrospectively analyzed the pre-operative screening for HIV, HBV, and HCV in all the patients undergoing oral surgery between April 2014 and March 2022. Blood samples were collected from the participating patients by venipuncture. Written informed consent was obtained from the patients and from the relatives of those under 20 years of age, prior to the test. If several tests were performed during the 6 months, only the first procedure was analyzed.

### 2.3. Screening Method

Each screening was evaluated using the ARCHITECT i2000SR Immunoassay Analyzer (Abbott Japan Co., Ltd., Tokyo, Japan) using chemiluminescent microparticle immunoassay. Each patient whose results exceeded the manufacturer’s recommended viral load thresholds (hepatitis B surface antigen (HBsAg) quantification < 0.05 IU/mL (IU = International Unit), HCV antibody quantification < 1.0 S/CO (S/CO = Sample RLU/cut off), or HIV antigen antibody (<1.0 S/CO)) using a rapid test kit was referred to a hepatitis specialist or hematologist for hepatitis and HIV, respectively. Hepatitis and HIV infections that were self-reported by the patients were assessed from the questionnaire. Prior to the medical interview with the oral surgeon, the patient or guardian filled out the questionnaire. The oral and maxillofacial surgeons did not directly ask questions, unless hepatitis or HIV was mentioned in the questionnaire.

### 2.4. Cost-Effectiveness Analysis

For the cost-effectiveness analysis, we tracked the cost of HBV, HCV, and HIV screening tests on a patient-by-patient basis. The cost of identifying the patients with active or cleared viral load was calculated by multiplying the cost of rapid testing by the number of patients and dividing it by the number of positive patients. This calculated the cost of universal screening. That is, it was compared to the estimated cost of allowing each infection to pass undiagnosed by preoperative screening.

### 2.5. Statistical Analysis

The self-reporting rate was the percentage of patients who had been treated or diagnosed with each infectious disease and who self-reported them on the questionnaire. The rate of detection of patients with untreated infectious diseases was evaluated by the percentage of untreated patients among the positive patients. All data are presented as means and their standard deviations. For group comparisons, the Mann–Whitney U test was used for continuous variables and the Chi-squared test or Fisher’s exact test were used for categorical variables. The relationship between oral diseases and the prevalence of viral hepatitis and HIV were analyzed by logistic regression analysis, with control for age and sex, using commercially available software (JMP 14.2 for Mac; SAS Institute Inc., Cary, NC, USA). Statistical significance was set at a two-tailed *p*-value < 0.05.

## 3. Results

### 3.1. Infectious Disease Test Counts and Positivity Rates

Of all the patients with oral diseases and conditions tested for HBV, HCV, and HIV, 62 out of 15,842 (0.39%) were positive for HBsAg and 153 out of 15,839 were positive for hepatitis C (0.97%). Ten out of 12,745 (0.08%) were HIV-positive (Table 1).

### 3.2. Patient Declaration Rate for Infectious Diseases

Among the patients who tested positive in the pre-operative screening test and who had been treated, the self-reported pre-operative positivity rate for HBV was 45.7% (26/41), for HCV, it was 50.4% (62/123), and for HIV, it was 87.5% (7/8) (Table 2).

### 3.3. New Detection Rate for Infectious Disease-Positive Patient

Among the patients who tested positive in the pre-operative screening test, the positivity rate of new patients with infectious diseases who had not been treated earlier was 33.8% (21/62) for HBV, 19.6% (30/153) for HCV, and 10% (1/8) for HIV (Table 3).

### 3.4. Detection Rate of Untreated Infectious Disease Patients by Infectious Disease Screening Test

Among the patients who underwent screening for infectious diseases, the positivity rate of new patients with infectious diseases who had not been treated was 0.0013% (21/15,801) for HBV, 0.0019% (30/15,809) for HCV, and 0.0001% (1/12,744) for HIV (Table 4).

### 3.5. Relationship between Sex, Age, and Infectious Diseases

Significantly more males than females were affected by HBV, HCV, and HIV (*p* value: HBV = 0.0043, HCV ≤ 0.0001, HIV = 0.0002). All the patients with HIV were male.

In terms of the relationship between age and infectious diseases, HBV and HCV affected significantly higher age groups (*p* value: HBV ≤ 0.0001, HCV ≤ 0.0001). Conversely, there was no significant difference with age between HIV with and without infection (*p* value: HIV = 0.7675) (Table 5).

### 3.6. Prevalence of Infectious Diseases, According to Various Oral Diseases

Most of the patients with oral diseases required tooth extraction. The highest proportion of infectious-disease-positive patients was 2.03% (4/193) for oral malignancies in HBV and 3.47% (7/195) and 0.67% (1/148) for inflammation in HCV and HIV, respectively (Table 6). The relative prevalence of HBV, HCV, and HIV infection was analyzed using logistic regression analysis. The odds ratios were assessed by adjusting for age and sex for HBV and HCV, and sex for HIV, based on tooth extraction, which was the most commonly performed oral surgical procedure. The odds ratios for various other oral diseases and conditions were not significant for any of the infections (Table 7).

### 3.7. Cost-Effectiveness Analysis

The total cost of screening for HBV, HCV, and HIV was 13,904,960 yen ($116,174.7), 16,630,950 yen ($138,591.3), and 14,274,400 yen ($118,953.3), respectively. A total of 15,842, 15,839, and 12,745 patients with HBV, HCV, HIV infections, respectively, were screened and the cost per positive result was 224,854.2 yen ($1873.8) for HBV (total HBV screening cost: 13,940,960 yen/62 positive patients), 108,699.0 yen ($905.8) for HCV (total HCV screening cost: 16,630,950 yen/153 positive patients), and 1,427,440 yen ($11,895.3) for HIV (HIV screening total cost: 14,274,400 yen/10 positive patients).

On the other hand, the screening cost for untreated patients was 663,855.2 yen ($5532.1) for HBV (total cost for HBV screening: 13,940,960 yen/21 untreated positive patients), 554,365 yen ($4619.7) for HCV (total cost for HCV screening: 16,630,950 yen/ 30 untreated positive patients), and 14,274,400 yen ($118,953.3) for HIV (total cost of HIV screening: 14,274,400 yen/untreated positive patient).

## 4. Discussion

In this study, we investigated the prevalence of HBV, HCV, and HIV in patients scheduled to undergo oral and maxillofacial surgery. The prevalence of HBV, HCV, and HIV was 0.39, 0.76, and 0.07%, respectively. Age was a significant factor affecting HBV and HCV, and males were affected significantly more by all three infections. The self-reported positivity rates of HBV, HCV, and HIV on the patient questionnaire were 63.4, 50.4, and 87.5%, respectively. Self-assessment using the questionnaire may be effective to some extent, but the accuracy was uncertain. It was also not cost-effective for screening untreated patients.

In our study, the prevalence of HBV, HCV, and HIV infection in the patients undergoing oral and maxillofacial surgery was found to be 0.39, 0.97, and 0.08%, respectively. Takata et al. [12] reported that the prevalence of viral hepatitis infection in oral and maxillofacial surgery patients was 2.1% for HBV and 5.8% for HCV. This prevalence was greater when compared to our results. Patients with impacted teeth and orthognathic surgery in the previous study [3] comprised 13.3% of the total number (587/4402). However, in our study, they accounted for 70.1% (11,100/15,842) of HBV infections and 69.1% (10,952/15,839) of HCV infections. Our study also showed that age and sex are significant factors affecting hepatitis [1]. This is consistent with the results of previous clinical studies.

Malignant oral tumors are generally observed in old patients, while jaw deformities and wisdom tooth extractions are often observed in young individuals. Differences in these results may be owing to differences in patient distribution.

No significant difference in the prevalence of HBV, HCV, and HIV infections was apparent between patients with different categories of oral diseases or conditions, even after adjusting for age. Therefore, predicting viral infections pre-operatively based on the type of oral disease is challenging. This result is different from the report by Takada et al. [12], wherein benign tumors in HBV and malignant tumors in HCV showed high odds ratios for age-adjusted oral disease-specific infections; however, the distribution of patients according to the study design had an effect, as well as other entanglements, which could be influenced by the factors. Our study could be useful considering previous studies.

The self-reported screening of patient questionnaires is cost-effective and non-invasive. In our study, the self-reported positive infection rates from pre-operative questionnaires were as follows: HBV, 63.4% (26/46); HCV, 50.4% (62/123); HIV 87.5% (7/8). These results indicate that patient self-reporting is an inadequate screening method. In a Japanese study by Nagao et al. [13], 209 patients being treated for liver disease responded to a questionnaire to determine if they had disclosed their condition personnel in dental clinics. It was found that 59.8% always disclosed, 12% sometimes disclosed, and 28.2% never did. The main reason for non-disclosure was that the dental practitioner did not ask if the patient had a systemic illness. Other reasons included fear of negative reactions from dental health care workers and the belief by infected individuals that hepatitis was unrelated to dental care. In the field of dentistry and maxillofacial treatment, it has become clear that information about infection may not be accurately conveyed by the questionnaire alone.

The prevalence of HIV in our study was 0.08%, which was lower than the prevalence (1.1%) shown in a similar study by Dreyer et al. [14]. There was no significant difference in the combined prevalence of HIV infection between patients with different oral diseases or ages. In our study, patients self-reported a positive HIV infection rate of 87.5% (7/8). This result is relatively high; however, it should be taken into account that the number of patients enrolled in our study was very small. Considering the changes in the relationship between patients with HIV and dental practitioners, studies in the 1990s tended to withhold information about the HIV status from practitioners [15,16]. However, in recent years, some research has shown that patients are more likely to be comfortable discussing HIV with their dentists [17,18]. Similar results were obtained in this study. Regarding HIV screening, it will be necessary to carry out further research with a larger number of cases, taking into consideration the background of the times.

Screening for infectious diseases costs approximately $1900 for HBV, $900 for HCV, and $12,000 for HIV per positive result. Nussbaum et al. [19] reported that each positive HCV result during neurosurgery screening required approximately $3900. Screening also contributed to the elimination of health risks for surgical staff. Since the results of our study contributed to the detection of patients with infectious diseases at a much lower cost than the previous study [19], hepatitis screening was considered to be cost-effective for patients undergoing oral and maxillofacial surgery in Japan. On the other hand, HIV screening cost a lot when compared to a previous study [20]. An Indian study examining HIV screening and costs indicated it was $418.9 (screening patients = 4529 * $ 0.37 test costs/number of positive patients = 4) [20]. Although it is not possible to make a simple comparison because the cost of testing and the number of people living with HIV varies from country to country, the cost-effectiveness of the universal screening test for HIV does not seem to be very good in Japan. On the other hand, surgical treatment carries the risk of infection and should be assessed from the perspective of a healthcare professional. It is mentally reassuring for healthcare professionals to know the existence of prior infections through infectious disease screening in advance. In addition, it would be possible to respond promptly and reliably in the event of complications such as needle sticks; thus, it will be possible to accomplish smoother daily treatment. Therefore, for medical application, even less cost-effective tests may make sense.

Our research has some limitations. Firstly, it may contain false positives for patients with infectious diseases. All the screening-positive patients associated with this study were encouraged to have a consultation with a liver physician or hematologist. However, it is unclear whether all the cases were truly positive since the patients refused additional detailed tests, or there were cases that wanted to be seen outside our hospital. Since it is not the primary task of oral and maxillofacial surgeons to treat each infectious disease, it is clinically relevant to identify false positives as positives. Secondly, we did not actively interview patients who did not have an infectious disease mentioned on the questionnaire. If we had done so, it may have strengthened screening through more aggressive interviews. Overcoming this limitation requires more planned prospective studies that are conducted after educating dental and oral surgeons on how to interview patients with infectious diseases in advance.

## 5. Conclusions

In this retrospective study, we investigated the prevalence of HBV, HCV, and HIV in patients scheduled for oral and maxillofacial surgery. The prevalence of HBV, HCV, and HIV was 0.39, 0.76, and 0.07%, respectively. The self-reported positivity rates of HBV, HCV, and HIV on the patient questionnaire were 63.4, 50.4, and 87.5%, respectively. Self-assessment using questionnaires may be effective to some extent, but the screening accuracy was inadequate. Age was a significant factor affecting HBV and HCV, and males were affected significantly more for all the infections. Unfortunately, it was difficult to make an auxiliary diagnosis of infectious disease using the presence of oral diseases and conditions. The cost per positive result was useful for hepatitis, but less effective for HIV.

## Figures and Tables

**Table 1:** Infectious disease test counts and positivity rates.

	HBV	%	HCV	%	HIV	%
positive	62	0.39	153	0.97	10	0.08
negative	15,780	99.61	15,686	99.03	12,735	99.92
total	15,842		15,839		12,745	

HBV; hepatitis B virus, HCV; hepatitis C virus, HIV; human immunodeficiency virus.

**Table 2:** Patient declaration rate for infectious diseases.

	HBV	%	HCV	%	HIV	%
positive	26	63.41	62	50.41	7	87.50
negative	15	36.59	61	49.59	1	12.50
total	41		123		8	

HBV; hepatitis B virus, HCV; hepatitis C virus, HIV; human immunodeficiency virus.

**Table 3:** New detection rate for infectious-disease-positive patient.

	HBV	%	HCV	%	HIV	%
With medical treatment history	41	66.13	123	80.39	8	88.89
Untreated	21	33.87	30	19.61	1	11.11
Total	62		153		10	

HBV; hepatitis B virus, HCV; hepatitis C virus, HIV; human immunodeficiency virus.

**Table 4:** Detection rate of untreated infectious disease patients by screening test.

	HBV	%	HCV	%	HIV	%
Patients who have undergone examinations excluding treated patients	15,801		15,809		12,744	
Untreated	21	0.0013	30	0.0019	1	0.0001

HBV; hepatitis B virus, HCV; hepatitis C virus, HIV; human immunodeficiency virus.

**Table 5:** Relationship between sex, age, and infectious diseases.

	Sex			Age	
	male	female	*p* value	Average ± SD	*p* value
HBV					
positive	37	6588	0.0043	58.23 ± 15.41	<0.0001
negative	25	9192		40.13 ± 19.69	
HCV					
positive	90	6534	<0.0001	59.97 ± 18.78	<0.0001
negative	63	9152		40.02 ± 19.62	
HIV					
positive	10	5345	0.0002	42.20 ± 11.77	0.7675
negative	0	7390		40.34 ± 19.89	

SD; standard deviation; HBV; hepatitis B virus, HCV; hepatitis C virus, HIV; human immunodeficiency virus.

**Table 6:** Prevalence of infectious diseases, According to various oral diseases.

	HBV			HCV			HIV		
Oral diseases, conditions, or treatment	negative	positive	positivity rate (%)	negative	positive	positivity rate (%)	negative	positive	positivity rate (%)
Tooth extraction	10,670	36	0.34	10,622	78	0.73	8702	7	0.08
Dental implant	2630	11	0.42	2603	39	1.48	2023	1	0.05
Cyst, benign tumor	1358	5	0.37	1348	14	1.03	1037	1	0.10
Trauma	230	1	0.43	225	7	3.02	199	0	0
Alveolar surgery	212	3	1.40	212	3	1.40	183	0	0
Inflammation	198	2	1.00	195	7	3.47	148	1	0.67
Oral malignancies	193	4	2.03	195	2	1.02	183	0	0
Jaw deformity	118	0	0	118	0	0	110	0	0
Salivary gland surgery	86	0	0	85	1	1.16	73	0	0
Congenital anomalies	41	0	0	41	0	0	41	0	0
Maxillary sinus surgery	37	0	0	35	2	5.41	30	0	0
Temporomandibular joint surgery	7	0	0	7	0	0	6	0	0

HBV; hepatitis B virus, HCV; hepatitis C virus, HIV; human immunodeficiency virus.

**Table 7:** A logistic regression analysis comparing the prevalence of infectious diseases in oral diseases, conditions, or treatments, with the prevalence of patients undergoing tooth extraction.

Oral Diseases, Conditions, or Treatment	HBV (Adjusted for Age and Sex)		HCV (Adjusted for Age and Sex)		HIV (Adjusted for Sex)	
	Odds Ratio (95% CI)	*p* Value	Odds Ratio (95% CI)	*p* Value	Odds Ratio (95% CI)	*p* Value
Dental implant	1.977 (0.982–3.982)	0.056	1.297 (0.869–1.938)	0.204	1.665 (0.205–13.549)	0.634
Cyst, benign tumor	1.586 (0.616–4.083)	0.339	1.283 (0.719–2.287)	0.399	1.003 (0.123–8.168)	0.998
Oral malignancies	0.630 (0.212–1.868)	0.405	3.155 (0.759–13.118)	0.114	-	-
Alveolar surgery	0.633 (0.189–2.122)	0.459	1.518 (0.469–4.909)	0.486	-	-
Trauma	1.358 (0.184–10.049)	0.764	0.507 (0.215–1.195)	0.120	-	-
Inflammation	0.917 (0.212–3.966)	0.908	0.621 (0.275–1.406)	0.253	0.131 (0.016–1.078)	0.059
Jaw deformity	-	-	-	-	-	-
Salivary gland surgery	-	-	1.159 (0.157–8.561)	0.885	-	-
Maxillary sinus surgery	-	-	0.276 (0.063–1.213)	0.088	-	-
Congenital anomalies	-	-	-		-	-
Temporomandibular joint surgery	-	-	-	-	-	-

HBV; hepatitis B virus, HCV; hepatitis C virus, HIV; human immunodeficiency virus, CI: Confidence Interval.

## Data Availability

The datasets used and/or analyzed during the current study are available from the corresponding author upon reasonable request.
